# The Practice of Surgery

**Published:** 1873-04

**Authors:** 


*The Practice of Surgery.* By THOMAS BRYANT, F.R.C.S., Surgeon to Guy's Hospital. With five hundred and seven illustrations. Philadelphia: Henry C. Lea, 1873. Buffalo: T. Butler & Son.

The reputation of the author and his extended facilities for observation as Surgeon to Guy's Hospital, has caused the advent of his work on surgery to be awaited, by the profession, with no small measure of expectation and curiosity. The work as it is now placed before the profession will not, we think, disappoint those expectations; indeed, the author, in endeavoring to place in the hands of his readers a "manual," has, with a few exceptions, given them a treatise. The consideration of the diseases and surgery of the eye and ear, and of dental surgery, has been entirely omitted, and although it detracts in a large measure from the value of the work as a complete treatise, it in no wise prevents it from being what it claims to be, a Manual of the Practice of Surgery. As the author truly says, " to have given them in outline would have been to mislead." In the introductory chapter the author speaks of the mode of observation in surgical practice, and of the necessity and value of knowing how to observe. The different surgical affections and their treatment are considered in their appropriate order, and the directions for diagnosis and treatment arc clearly laid down and easily undeistood· Many of the operative procedures are either peculiar to the writer, or to English surgery, and although at variance with opinions held by American surgeons, are not advised by him without careful consideration and trial. He has not hesitated, like many English writers, to go out of his own country for facts, and due credit is given for nearly all important discoveries and operations emanating from American surgery. Some mistakes have naturally crept into his work, but they are of small importance. We hardly think that American operators will accord to British surgeons the credit of introducing Ovariotomy to the world, and the statement that Atlee generally employs the ecraseur in severing the pedicle will cause some surprise. The assertion that the preservation of the periostium is a matter of but little importance in exsections, must, we think, be received with some grains of allowance. The author has drawn largely on the rich store of specimens in Guy's Hospital to illustrate his subjects and has produced in this department almost an entirely original work. The observations of the writer are faithfully recorded, and his own views clearly set forth. We cannot but recommend the work to our readers, and are of the opinion that Bryant's Surgery is a book which no progressive surgeon can afford to be without.

